# Breakage of intramedullary femoral nailing or femoral plating: how to prevent implant failure

**DOI:** 10.1186/s40001-021-00630-7

**Published:** 2022-01-13

**Authors:** Henrik C. Bäcker, Mark Heyland, Chia H. Wu, Carsten Perka, Ulrich Stöckle, Karl F. Braun

**Affiliations:** 1 Department of Orthopaedic Surgery and Traumatology, Charité Berlin, University Hospital Berlin, Chariteplatz 1, 10117 Berlin, Germany; 2 Department of Orthopedics & Sports Medicine, Baylor College of Medicine Medical Center, Houston, TX USA; 3 Department of Traumatology, University Hospital Rechts Der Isar, Technical University Munich, Munich, Germany

**Keywords:** Femoral, Fracture, Implant, Failure, Complication

## Abstract

**Introduction:**

Intramedullary (IM) fixation is the dominant treatment for pertrochanteric and femoral shaft fractures. In comparison to plate osteosynthesis (PO), IM fixation offers greater biomechanical stability and reduced non-union rates. Due to the minimally invasive nature, IM fixations are less prone to approach-associated complications, such as soft-tissue damage, bleeding or postoperative infection, but they are more prone to fat embolism. A rare but serious complication, however, is implant failure. Thus, the aim of this study was to identify possible risk factors for intramedullary fixation (IMF) and plate osteosynthesis (PO) failure.

**Materials and methods:**

We searched our trauma surgery database for implant failure, intramedullary and plate osteosynthesis, after proximal—pertrochanteric, subtrochanteric—or femoral shaft fractures between 2011 and 2019. Implant failures in both the IMF and PO groups were included. Demographic data, fracture type, quality of reduction, duration between initial implantation and nail or plate failure, the use of cerclages, intraoperative microbiological samples, sonication, and, if available, histology were collected.

**Results:**

A total of 24 femoral implant failures were identified: 11 IMFs and 13 POs. The average age of patients in the IM group was 68.2 ± 13.5 years and in the PO group was 65.6 ± 15.0 years, with men being affected in 63.6% and 39.5% of cases, respectively. A proximal femoral nail (PFN) anti-rotation was used in 7 patients, a PFN in one and a gamma nail in two patients. A total of 6 patients required cerclage wires for additional stability. A combined plate and intramedullary fixation was chosen in one patient. Initially, all intramedullary nails were statically locked. Failures were observed 34.1 weeks after the initial surgery on average. Risk factors for implant failure included the application of cerclage wires at the level of the fracture (*n *= 5, 21%), infection (*n* = 2, 8%), and the use of an additional sliding screw alongside the femoral neck screw (*n* = 3, 13%). In all patients, non-union was diagnosed radiographically and clinically after 6 months (*n* = 24, 100%). In the event of PO failure, the placement of screws within all screw holes, and interprosthetic fixation were recognised as the major causes of failure.

**Conclusion:**

Intramedullary or plate osteosynthesis remain safe and reliable procedures in the treatment of proximal femoral fractures (pertrochanteric, subtrochanteric and femoral shaft fractures). Nevertheless, the surgeon needs to be aware of several implant-related limitations causing implant breakage. These may include the application of tension band wiring which can lead to a too rigid fixation, or placement of cerclage wires at the fracture site.

## Introduction

While annual hip fracture incidence declined from 600/100,000 to 400/100,000 person-years from 1996 to 2006, the rarer femur fractures did not decline with incidence rates for subtrochanteric and femoral shaft fractures each below 20 per 100,000 person-years [1]. This incidence of femoral shaft fractures (18.2 per 100,000 person-years [2, 3]), and subtrochanteric fractures (ranging from 10.8 to below 20 per 100,000 person-years) shows a combined incidence below 30 per 100,000 person-years, so this type of fracture is much less common than proximal femur (hip) fracture [4, 5]. Treatment options include plate or intramedullary fixation. AO (Arbeitsgemeinschaft für Osteosynthesefragen, Switzerland) principles emphasise a biologically friendly fixation strategy, anatomical reduction, and adequate micromotion and stiffness at the fracture site to promote healing [6]. The restoration of length, alignment and rotation is emphasised in the fixation of femoral fractures [7].

For treatment, intramedullary nails are less invasive with a lower risk of complications by avoiding damage to the periosteal circulation. In addition, these enable elasticity and, therefore, micro-movement to the facture site. Hereby, the widest and longest nails should be used to achieve a close fit between implant and bones including rotational and lateral stability [8, 9].

Plate osteosynthesis, on the other hand, requires a more open approach, either less invasive or open, to allow an anatomical reduction. It is easier to control the fracture and reduce it through direct visualisation; however, the periosteal circulation may be reduced with the plate pressed onto the bone. Plate osteosynthesis is indicated, especially for comminuted fracture, or in the presence of a narrow femoral shaft, and also for periprosthetic fracture. Besides offering a stable fixation, some stress shielding to the bone occurs, which may induce insufficient strain to the fracture site and, therefore, cause an inadequate healing response associated to non-union, eventually leading to implant failure [10–12].

Common complications after fixation include bleeding, malalignment and infection. Modern fixation hardware are associated with low implant failure rates [13]. Intramedullary femoral nails can fail at the proximal screw aperture, as seen in revision nail fixations [14]. In contrast to plate osteosynthesis, intramedullary nailing reaches superior load-to-failure as the implant is closer to the central weight-bearing axis to the femur, which reduces the bending stress by up to 30% [15]. Furthermore, a dynamic stabilisation can be performed to improve compression. On the other hand, disadvantages include the unsuitability of intramedullary for periprosthetic fractures [16]. Plate osteosynthesis has the advantage of allowing compression and direct reduction through the plate in femoral shaft fracture [17, 18].

This study aims to analyse the different causes of implant failure after femoral fracture fixation, including demographics, operative techniques, fracture pattern, the use of cerclage wires, and, if available, histology samples.

## Methods

A retrospective study was performed at a major level 1 trauma centre including patients between 2011 and 2019 after obtaining internal review board approval. A fellowship-trained orthopaedic trauma surgeon analysed the internal trauma registry for femoral implant failure and reviewed patient charts as well as plain radiographic imaging. Inclusion criteria consisted of patients aged 18 years or older, suffering from either femoral nail or plate implant failure after per-/subtrochanteric or femoral shaft fracture. Implant failure was defined as either intramedullary nail or plate breakage. Exclusion criteria included simple screw failures as well as the dislocation of implant without failure.

Data on demographics, gender, age and comorbidities were collected. Furthermore, we were interested in time to failure after initial implantation, usage of implant, mechanism of injury (e.g., high versus low energy accidents), location of implant failure and the type of screw fixation (e.g., static versus dynamics stabilisation). Postoperative quality of reduction was defined according to the Lowell’s Criteria in anteroposterior plain radiography which included anteroposterior alignment, displacement and angulation of the two fracture segments [19, 20]. In addition, we were interested in the application of cerclage wires, including the distance between cerclage wires if more than one was applied as well as the distance between A. circumflexa femoris and cerclage wire. In all patients, biopsies were obtained during revision surgery including prolonged microbiology cultures and histology.

For statistical analysis, IBM SPSS and Origin ANOVA was used. Mean and standard error of the mean were calculated assuming the normal distribution of data.

## Results

In total, 37 patients with implant failures were found. Twelve were excluded due to injuries in other extremities including the tibia, fibular, humerus, distal radius, clavicular, pelvic plates, and dorsal spondylodesis. In addition, one patient presented with an intramedullary tibial nail failure after a high energy fall, leaving only 24 patients for inclusion. Patients presented with plate breakage in 54.2% (*n = *13/24), and intramedullary nail failure in 45.8% (*n = *11/24). The overall mean age at time of implant failure was 65.7 ± 15.1 years. Gender was equally distributed at 50.0% between males and females.

### Nail implant failure

In the intramedullary nail failure group, 63.6% (*n = *7/11) were males and the mean age was 68.2 ± 13.5 years. In this cohort, two patients suffered from diabetes, and two were immunocompromised—one from HIV and one from prednisolone therapy. Furthermore, two people were smokers. Five patients in the IM group were tumour patients (45.5%; 5/11%): two underwent surgery for prophylactic tumour stabilisations (*n = *2/11), and three patients received nailing as a fracture treatment—two with prostate cancer and one diffuse large B-cell lymphoma.

**Fig. 1:**
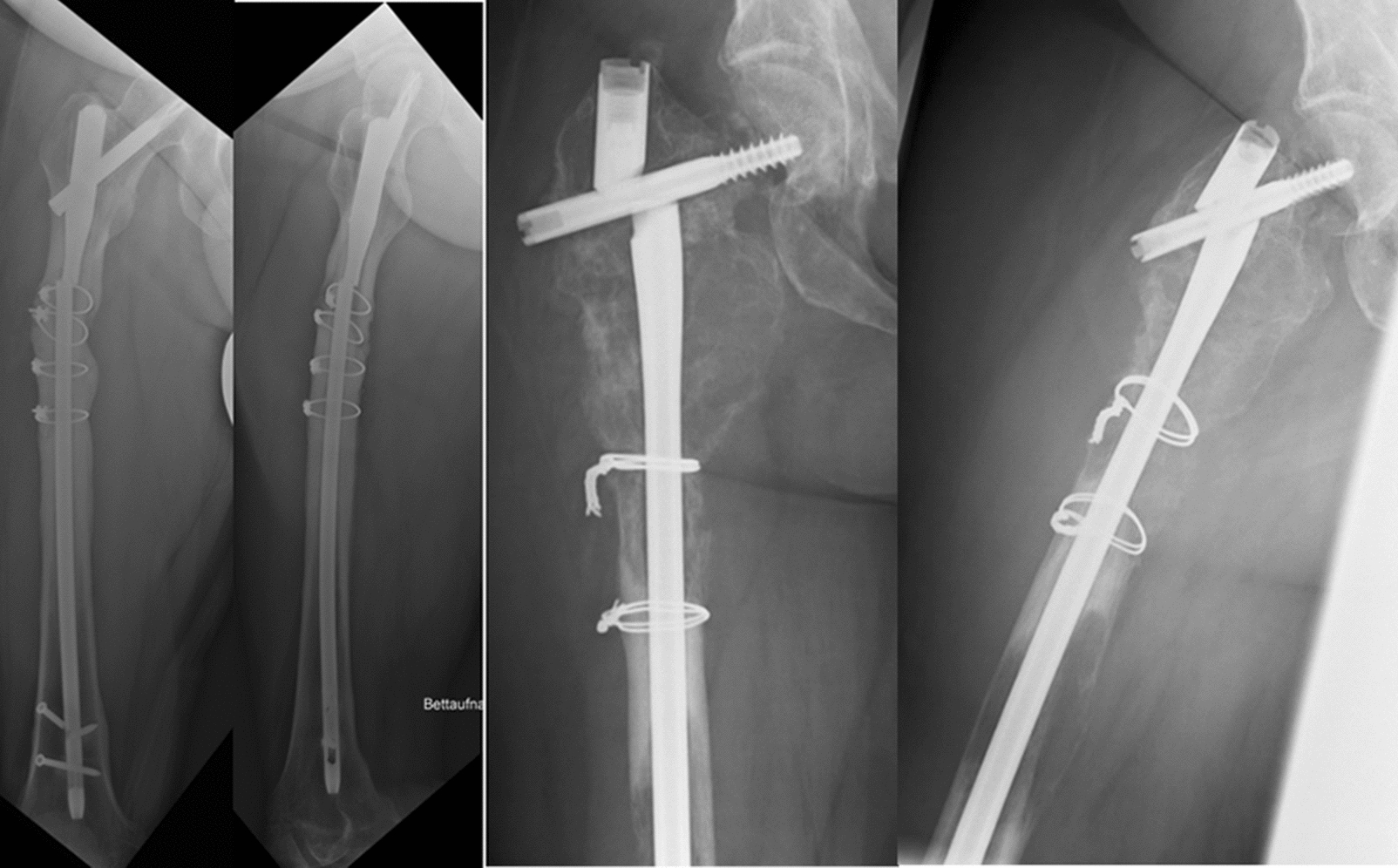
Two cases of intramedullary implant failure (**A**) cerclage migration as placed at the fracture site (**B**) at head screw with adequate placement of doubled wire cerclage and distance more than 5 cm

The femoral nail breakage occurred at a mean of 36.7 ± 20.3 weeks after initial surgery. All patients presented with sudden onset of pain in the hip, without any trauma. The Synthes proximal femur nail (PFN) was applied in 8 cases (72.7%; *n = *8/11), Stryker Gamma nail in two cases (1.8%; *n = *2/11) and in one case the nail type was not mentioned (Fig. 1). In all cases, an unreamed long femoral nail was applied with a diameter of 10 mm. The minimum inner cortical diameter of the femoral shafts was 16.1 ± 2.7 mm. All nails except one were locked statically (90.9%; *n = *10/11). Cerclages were applied in 6 patients. In 4 patients, a single Synthes cerclage wire system was applied, whereas either a double cable wire configuration or a single cable wire was used in the remaining two. In five of these cases, more than one cerclage wire was used. The distance between two cables was 28.9 mm on average (range from 17 to 54 mm). The most proximal cerclage wire was 24.3 mm on average (range from 0 to 52 mm) away from the anatomical location of the A. circumflexa femoris anterior. In this cohort, subtrochanteric fracture (54.5%; *n = *6/11) was the most common, followed by two pertrochanteric (18%; *n = *2/11) fracture patterns, one mid shaft fracture (9%; *n = *1/11) and one intertrochanteric fracture (9%; *n = *1/11). In addition, two patients underwent prophylactic tumour stabilisation with a MIREL score of 9—one for metastasis of melanoma and one for breast cancer.

The initial quality of reduction was anatomic in four cases. In 4 cases, a slight displacement of 7 mm and one case of 8 mm lateralisation was observed on the anteroposterior plain radiographic imaging. In two subjects, no postoperative X-rays were found.

The most common location for implant failure is between two cerclages, as seen in two cases. Breakage was seen at the location of the cephalomedullary screw in three subjects. In three cases, failure sites were close to the most proximal cerclage. In tumour patients, failure occurred at the site of the osteolysis and melanoma metastasis. In those cases, where a cerclage fixation was performed, a dislocation in the fracture was seen (80.0%; *n = *4/5) and non-union was observed radiographically in all cases.

At the time of implant failure, the mean serum C-reactive protein was 42.5 ± 68.6 mg/dl and a mean serum white blood cell count of 9.6 ± 3.4 G/l was reported. Histology samples were obtained in two cases which showed non-union without any signs of malignancy or infection. For microbiological cultures, samples remained sterile in 72.7% (*n = *8/11); in two patients, the growth of either *Staphylococcus aureus* (1.8%; *n = *2/11) or *Streptococcus constellatus* (0.9%; *n = *1/11) was present. In addition, sonication revealed *Streptococcus constellatus* in one case, and cutibacterium acnes in another.

### Plate implant failure

**Fig. 2:**
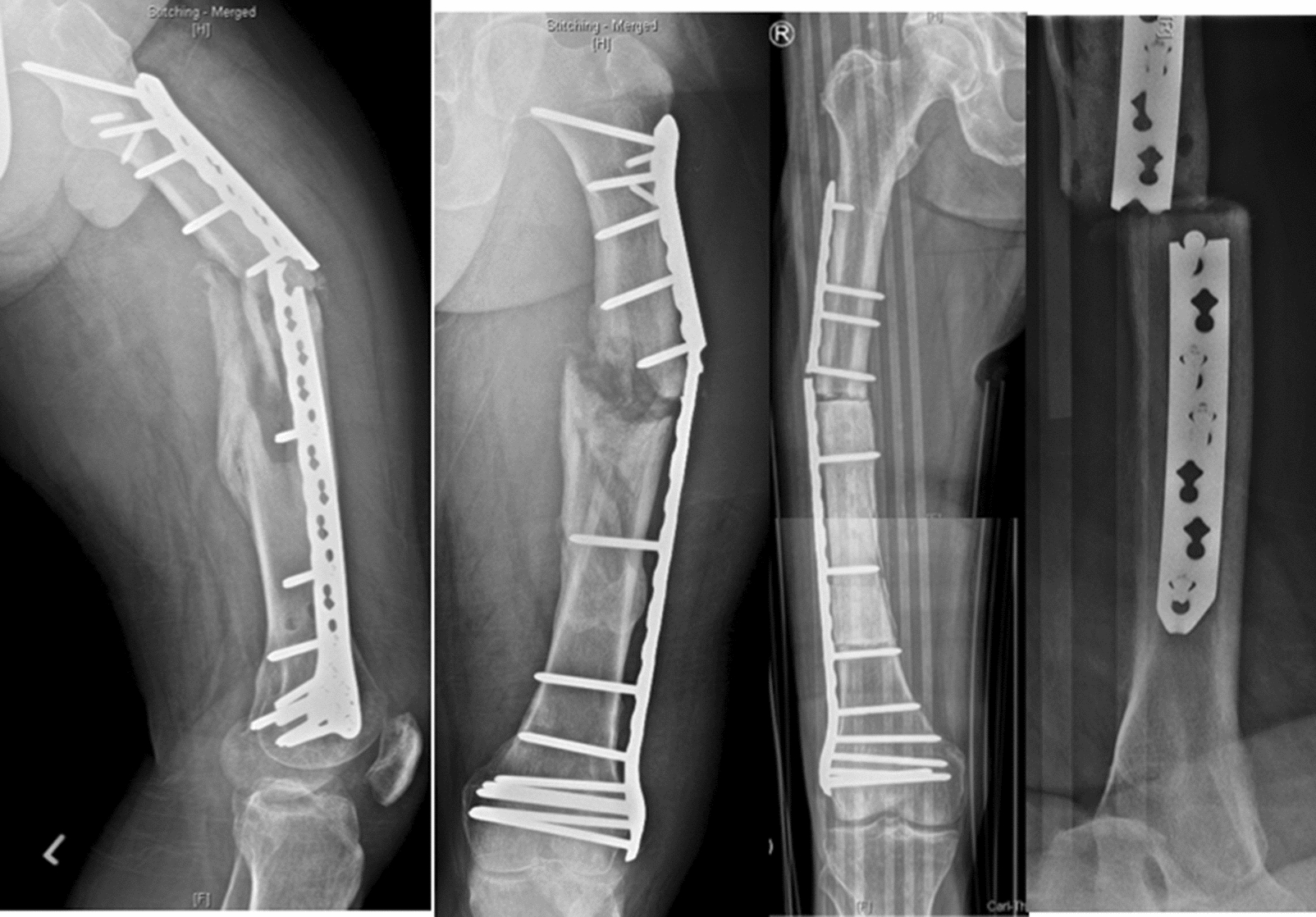
Two cases after plate fixation and implant failure with screws close to the fracture site

In the plate osteosynthesis group, the mean age was 65.6 ± 15.0 years at the time of hardware failure. Females made up 61.5% (*n = *8/13) of this group. The mean time between initial surgery and implant failure was 21.1 ± 18.9 weeks. In most cases, a Synthes LISS LCP femur plate made of surgical steel was used (*n = *12/13). In one patient, a DCP plate was used, and breakage was observed (Fig. 2). In total, plate fixation was utilised for two C3 midshaft fractures, three C3 distal shaft fractures, two prophylactic tumour stabilisations with a MIREL score of 9 (NSCLC), and one clear cell renal cell carcinoma. In addition, plate fixation was also selected for the treatment of two Vancouver C fractures, one interprosthetic fracture, one case of third degree (3°) open C3 femoral shaft fracture, and one subtrochanteric fracture. Locking screws were placed in most cases (92.3%, *n = *12/13), while the screws were placed in a nonlocking manner in only one patient. The distance between screw placements was 63.8 ± 52.0 mm and the inner cortical diameter was 16.4 ± 3.3 mm on average. For risk factors, five patients were immunosuppressed with prednisolone (60%; *n = *3/5), one was exposed to chemotherapy (i.e., mitomycin) therapy, and one sustained 3° open femoral fracture. Furthermore, one patient suffered from both diabetes mellitus and periprosthetic knee joint infection 1 year prior. No smokers were identified in this cohort. All except one patient suffered from low energy trauma.

In all cases, open reduction was performed through the sub-vastus approach and the quality of reduction was anatomic in 5 patients (38.5%; *n = *5/13), and minor displacement was seen in three patients ranging between 3 and 7 mm on plain X-ray. Reduction was valgus in three cases, one in varus, and one had a loss of length. For intramedullary failures, non-union was identified in every patient. In three subjects, a single-looped cerclage wires system (Synthes) was applied with a mean distance of 33.5 mm between them. The typical location of implant failure was at the site of fracture close to or with a screw placed inside the fracture (53.8%; *n = *7/13). In those cases, where cerclage wires were applied, the implant failure occurred at a location close to them in 23.1% (*n = *3/13) of cases with cerclage dislocation. In the remaining cohort, plate breakage was observed for the fixation of an interprosthetic fracture between knee and hip prostheses (*n = *2/13; 15.4%), or a progressive osteolysis due to clear cell renal cell carcinoma (7.7%, *n = *1/13). The major predisposing factor was the placement of screws in almost every screw hole. The mean serum C-reactive protein was 24.4 ± 33.7 mg/dl and the white blood cell count was 8.4 ± 1.8 G/dl for the plate osteosynthesis group. Histology revealed infection in two cases, whereas microbiology cultures showed the growth of *Staphylococcus epidermidis* in two cases and *Staphylococcus aureus* in one. Furthermore, sonication revealed the growth of *Staphylococcus hominis* in one of the three cases.

All demographics are illustrated in Table 1.

**Table 1:** Demographics and anatomical variations between nail and plate fixation groups

	Nail fixation	Plate fixation
No. of patients	11	13
Gender (male) in no. (%)	7 (63.6)	8 (61.5)
Mean age (years)	68.2 ± 13.5	65.6 ± 15.0
Duration between initial surgery and failure (weeks)	36.7 ± 20.3	21.1 ± 18.9
Indication		
Fracture	9	8
Tumour stabilisation	2	2
Periprosthetic fracture	0	3
Comorbidities		
Immunosuppression	2	5
Diabetes mellitus	2	1
Cancer	3	1
Inner cortical diameter (mm)	16.1 ± 2.7	16.4 ± 3.3
Cerclage (no.)	6	3
Distance to A. circumflexa femoris anterior (mm)	24.3 (range 0–52)	N/A
Distance between cerclages (mm)	28.9 (range 17–54)	33.5 (range 25–44)
Distance between screws (mm)	N/A	63.8 ± 52.0
C-reactive protein (mg/dl)	42.5 ± 68.6	24.4 ± 33.7
White blood count (G/l)	9.6 ± 3.4	8.4 ± 1.8

*N/A* not applicable, *no *numbers, *mm* millimeter

## Discussion

This study outlines the risk factors for the failure of intramedullary as well as plate fixation of the femoral fracture. A trauma database was searched, and clinical charts were reviewed.

These risk factors can be classified into level of trauma, technical factors and biological factors. When looking for the level of trauma and accidental loads, this depends upon individual factors including initial weight bearing after surgery, body mass index, the tendency of falling and involuntary muscle spasms, especially in elderly patients. Hereby, excessive forces beyond the ultimate loads of the fixation strength may act on the implant, causing breakage. Therefore, surgeons should choose the implant as well as the type of surgery according to patient demographics and loading expectations. This may include the usage of thicker implants, stiffer materials, double plating or even a combination of nailing and plating.

More importantly, the type of surgery and excessive implants have to be chosen thoroughly. This includes the length of implant, placement of cerclage wires, locking attachment plates or the necessity of lag screws or narrowly placed screws [7, 21] which may cause local stress concentrations [22–24]. Biomechanical investigations showed the weakest spot of the nail to be at the lag screw hole. Forces greater than 1800 N can place the implant at a high risk of failure, especially when drilling off target [25]. Additional weak spots include distal shaft screw holes, where the diameter of the nail is reduced. This also requires an accurate screw placement in either the static or dynamic locking hole to minimise risk [26, 27]. Other factors include the diameter of the femoral nail, as well as a bicortical placement of the distal locking screws. For the placement of cerclage wires, this should be placed from the posterior intermuscular septum at the linea aspera, taking care to avoid a superficial and deep femoral artery. For the distal quarter of the femur, the tip of the cerclage wire passer should be close to the posteromedial and posterior cortex of the femur to prevent injury to the femoral arteries and the sciatic nerve [28]. Other vessels at risk are perforating arteries [29]. An animal study showed a significant decrease in blood circulation in the area between cerclages, raising concerns for non-union [30]. If cerclage wires were placed with a 5 cm space between them, at least one perforator artery was compromised in all cases [31, 32]. Besides poor placement, loose-lock stability can be compromised by surface resorption. One study, however, shows that well tightened cerclages demonstrated in-growth due to callus formation [33]. This includes placement in the fracture line, where it may cause malreduction, leading to instability and, therefore, non-union. Findings suggest that cerclages should be placed at least 1 cm from the tip of the single fracture fragment, especially in oblique fractures. Cerclage wires should be avoided in comminuted and butterfly fracture patterns [24, 33, 34]. Mechanical failure can be avoided by placing a 1 mm cerclage in a mechanically stable region, using a double-looped wire cerclage with a simple symmetrical twist between four and eight times [35, 36]. An increase in wire diameter by 50% increased the load to failure by up to 169% in a single cerclage wire, and up to 300% when doubly wired [37]. In those cases, there is concern for pressure-induced bone necrosis [33, 38]. Furthermore, it is important to be careful during insertion to avoid any damage and notch factors have to be considered which may cause stress-risers, peri-implant fracture and implant fatigue [39]. In plate osteosynthesis, modern plates have limited contact with bone, thus reducing contact pressure and minimising effects on bone blood supply. This enabled a significant decrease in contact area along the interface, to prevent pressure-induced necrosis and subsequent porosis [40]. For bone healing, either absolute or relative stabilisation is required to facilitate primary or secondary bone healing, respectively. In secondary healing, micromotion is required, especially in an axial direction [41]. Therefore, it is important to understand the biomechanics and mechano-biology of the fixation type and implant used. This may also refer to the implant design which could be unsuitably flexible or stress-sensitive.

On the other hand, the biological risk factors including co-morbidities have to be diminished, which requires the fracture pattern, subsequent to an anatomical reduction, to ensure vascularisation, avoid intraoperative fractures and minimise the risk of infection by the adoption of the perioperative antibiotics or, if required, local antibiotics. Comminuted factures can be exposed to substantial stress [14]. In specific cases, augmented biological devices should be used, such as the local application of growth factors, cell therapies or scaffolds.

In our cohort, implant failure occurred, especially in comminuted fractures. In principle, these fracture patterns are already predisposed for delayed or non-union, which was noted in the cases. Cerclage wires were required to achieve a sufficient anatomical reduction.

Besides the patients’ individual, technical and biological risk factors, the life expectancy needs to be considered, especially when stabilising osteolysis, including metastasis and tumours. The longer the lifespan (> 1 year), the more rigid the implant has to be since implants are not indicated for too many repetitive loading cycles. Therefore, the nail thickness, material composition or the use of double plating along with the combination of nail and plate osteosynthesis have to be chosen based upon the individual requirements. In a few cases, the implantation of an endoprosthesis such as individual prosthesis or total femur as well as the application of bone cement (PMMA augmentation) could be an alternative [42].

## Conclusion

Fracture fixation is complex and requires some understanding of anatomy, physiology, and biomechanics to achieve union and avoid implant failure. Although implant failure is infrequent, it can have devastating consequences. Therefore, surgeons should minimise potential risk factors by choosing the appropriate implant for a specific patient.

## Data Availability

All data are published in this manuscript.
